# Microbiome and Metaproteome of Craniofacial Implant Regions in Health and Disease

**DOI:** 10.1111/odi.15398

**Published:** 2025-06-02

**Authors:** Daniela Lattuf Cortizo, Renato C. V. Casarin, Hélvis E. S. Paz, Camila S. Stolf, Mabelle F. Monteiro, Mônica T. V. Labate, Márcio Z. Casati, Luciano Lauria Dib

**Affiliations:** ^1^ School of Dentistry, Dental Research Division Paulista University (UNIP) São Paulo Brazil; ^2^ Division of Periodontics, Department of Prosthodontics and Periodontics, Piracicaba Dental School State University of Campinas (UNICAMP) São Paulo Brazil; ^3^ Division of Periodontics, School of Dental Medicine University at Buffalo Buffalo USA; ^4^ Laboratory “Max Feffer” of Genetic of Plants, Department of Genetics, Escola Superior de Agricultura “Luiz de Queiroz” University of São Paulo São Paulo Brazil

**Keywords:** craniofacial prosthesis, head and neck neoplasms, host–microbial interactions, proteomics

## Abstract

**Background:**

Craniofacial defects from cancer surgery led to functional, aesthetic, and psychological challenges. Rehabilitation with craniofacial implants addresses these issues by improving prosthesis retention through osseointegration and providing predictable cosmetic results. However, maintaining a healthy transcutaneous region is essential for implant longevity.

**Objective:**

Evaluation of the microbial community and host response around extraoral implants.

**Methods:**

In an intrasubject control study design, 12 cancer patients who had undergone oculofacial rehabilitation with implant‐supported prostheses were included. Biofilm and peri‐implant fluid samples were collected from the transcutaneous region of healthy and diseased implants. Microbiome profiling was conducted through DNA sequencing of the V3–V4 region of the 16S rRNA gene, and proteome analysis was performed using liquid chromatography–tandem mass spectrometry.

**Results:**

Differentially abundant species were observed, with *Streptococcus intermedius* being the most abundant in diseased areas, followed by *Corynebacterium diphtheriae* and *Prevotella bivia* . Metaproteomic analysis revealed distinct protein expression patterns between the groups, with increased activation of proinflammatory responses and inactivation of anti‐inflammatory responses in the diseased group.

**Conclusion:**

The study demonstrated an increased abundance of pathogenic bacterial community accompanied by an imbalanced immune response, thereby highlighting host–microbial factors that can influence the success of osseointegration and facial rehabilitation.

## 1 Introduction

The treatment of malignant tumors in the craniomaxillofacial region often involves extensive surgery and radiotherapy, resulting in partial or total loss of craniofacial tissues, which can impact patients both functionally and cosmetically. Craniofacial implants (CI), titanium screws affixed to the facial bone, have become a preferred option for retaining facial prostheses and are often used reliably in head and neck cancer patients (Moore et al. 2019). The osseointegration process provides superior prosthesis retention in cancer patients compared to other retention methods (Cortizo et al. 2015). Consequently, maintaining the health of the transcutaneous region of CI is essential for implant longevity, as most patients have reduced bone quantity and quality, low tissue vascularization, and associated comorbidities from chemotherapy and radiotherapy, all factors influencing rehabilitation success (Cortizo et al. 2015; de Souza et al. 2020; Dos Reis et al. 2017).

However, similar to dental implants, biofilm accumulation at the abutment–implant interface and in the gap between the skin and the metal prosthetic retention structure—often due to insufficient hygiene—can lead to tissue crust formation around implants. This buildup may cause skin reactions, local inflammation, pain, and, ultimately, implant loss (Verheij et al. 2016). Additionally, severe peri‐implant soft tissue reactions in oncology patients, if untreated, can have systemic consequences (Brånemark et al. 1977). Although essential for preventing complications, studies on the etiological factors of dysbiosis in this specific region remain limited. Evidence from studies on facial prostheses suggests that skin occlusion by prostheses may create a niche for opportunistic pathogens, such as *Staphylococcus aureus* (Ariani et al. 2012). The bacterial community, primarily colonized by *S. aureus* and *Staphylococcus epidermidis* , has already been associated with percutaneous inflammation at the transcutaneous abutment site (Johansson et al. 2021). Additionally, given that inflammation is not always clinically detectable in implant‐retained facial prostheses, it is well documented that implants present biological drawbacks such as a reduced physical barrier against bacterial invasion into the submucosal tissue (Corrêa et al. 2019). Inflammatory cells, including peripheral monocytes that differentiate into macrophages and multinucleated cells, populate the blood clot and simultaneously trigger an inflammatory response at the implant surface. Additionally, titanium is a potential modulator of macrophage polarization, which may favor local inflammation (Zhang et al. 2019).

Since the transition from healthy to diseased implants is characterized by host inflammation in response to a dysbiotic biofilm (Corrêa et al. 2019), it is essential to understand the microbiological and inflammatory profiles around the transcutaneous region of CI, especially given the challenges in clinical diagnosis. High‐throughput techniques, such as microbiome and proteome analysis, can provide new biological insights into disease susceptibility by revealing immunoinflammatory and microbiological patterns (Belibasakis et al. 2019). To date, the microbial community around CI has not been characterized. Therefore, this study aimed to investigate the microbiological and proteomic profiles associated with soft tissue reactions in the transcutaneous region of CI in cancer patients to identify molecular signatures of health and disease in this population.

## 2 Methods

### 2.1 Study Design and Patient Selection

The study was designed as an observational, case–control, intrasubject control design involving head–neck cancer patients who had undergone orbital resection. This study was approved by the Ethical Committee in Research of Paulista University (CAAE: 36534720.6.00005512), following the principles of the Declaration of Helsinki and the STROBE guidelines for observational studies.

**FIGURE 1:**
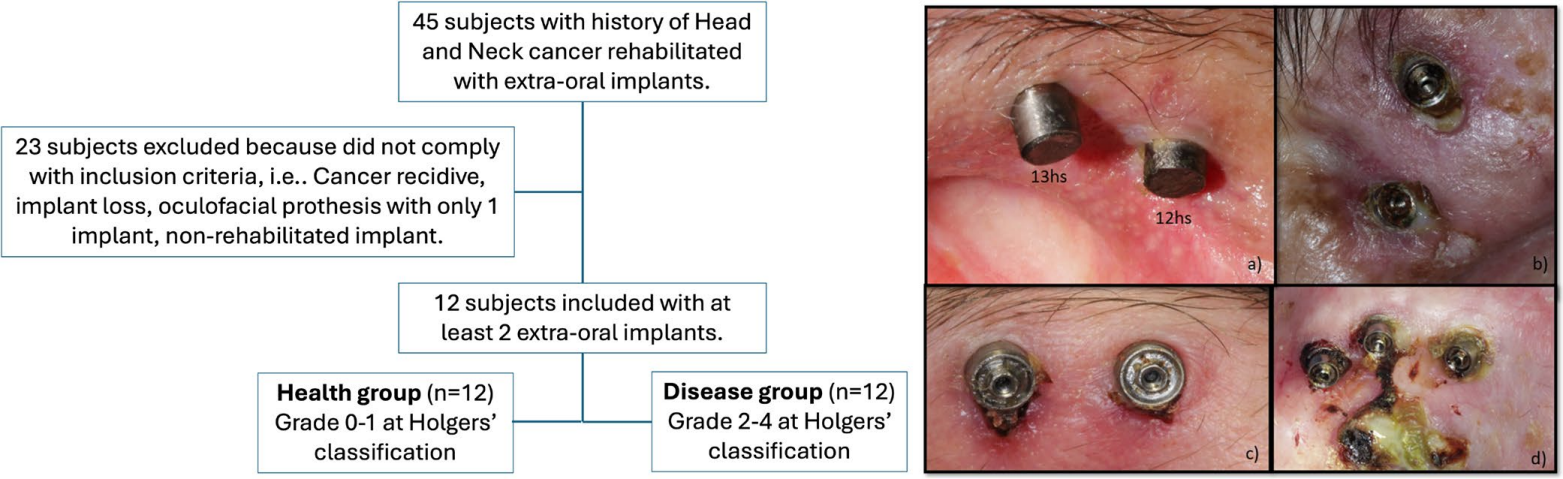
Flowchart of the study (right); Clinical aspects of the participants included in the study, showing: (a) the aspect of TRCI in both health (H) and disease (D) within the same individual; (b, c, d) different clinical cases and various aspects of skin reactions in the transcutaneous region of extraoral implants, ranging from inflammation to infection. In (c), crust formation is observed, and in (d), the progression of infection to the orbital cavity (left).

From 45 patients evaluated, 12 patients with implant‐supported oculofacial prostheses anchored by at least two implants were recruited (Figure 1). The inclusion criteria were to be an oncological patient with at least two osseointegrated extraoral implants that were used to retain an oculofacial prosthesis, with one implant being in a healthy condition and the other in a diseased condition, and who agreed to take part in the study. The Classification by Holgers et al. (Holgers et al. 1988) was used to classify the implants. Holgers' classification is a widely used system for assessing soft tissue reactions around extraoral implants, particularly in bone‐anchored hearing aids and craniofacial prostheses. This system categorizes peri‐implant skin reactions into five grades, aiding in clinical evaluation and management: *Grade 0*: No irritation or skin reaction. The skin around the implant remains intact, showing no signs of redness, swelling, or discharge; *Grade 1*: Slight redness (erythema) without swelling or the presence of granulation tissue. This stage is generally mild and does not require intervention; *Grade 2*: Redness and slight swelling, with possible minor discharge or granulation tissue formation. This stage may require topical treatment to prevent further progression; *Grade 3*: Significant redness, swelling, and granulation tissue formation, often accompanied by discharge. Medical intervention, such as antibiotics or local debridement, may be necessary; *Grade 4*: Severe soft tissue infection with extensive granulation tissue, swelling, and discharge. In some cases, this stage may lead to implant loss and require surgical intervention. A clinical examination of the transcutaneous region of CI (TRCI) for each implant was conducted by an experienced oral oncologist (L.L.D.). The implants were allocated into health group (*n* = 12), with TRCI classified as Grades 0 and 1, representing none or very mild signs of inflammation, and disease group (*n* = 12), with TRCI classified as Grades 2, 3, and 4, representing a higher degree of inflammation (Figure 1). If the patient presented more than two implants eligible for the study, the most distant implants presenting the inclusion criteria to each group were included in biofilm and peri‐implant crevicular fluid collection.

### 2.2 Sample Collection and Processing

Biofilm and peri‐implant fluid samples were collected from the TRCI region using sterile paper point strips and sterile PerioPaper strips (Oraflow, Plainview, NY, USA). First, six paper points per implant were carefully inserted into the space between the skin and the implant and left in place for 30 s, avoiding mechanical irritation of the transcutaneous region. Paper points were pooled into the same microcentrifuge tube for microbiome analysis. Afterward, at the same implant, six strips per implant were inserted and left in place for 30 s, avoiding mechanical irritation of the transcutaneous region. Strips were collected and pooled into the same tube for proteomic analysis. The strips/paper points contaminated by blood were excluded and a new collection was done after 1 min. Biofilm samples were stored at −20°C until microbiome analysis, while PerioPaper strips were stored at −80°C until proteomic analysis.

The biofilm from the paper point strips was removed from the paper cones by adding 200 μL of Tris‐EDTA buffer and vortexing for 1 min. DNA was then isolated using the PureLink Microbiome DNA Purification Kit (Thermo Fisher), following the manufacturer's specifications, and stored at −20°C until the amplification and sequencing protocol.

For peri‐implant fluid, the samples were recovered from the PerioPaper by solubilizing them in 400 μL of TCT buffer (7 M urea, 2 M thiourea, 10 mM DTT, and 0.1% (v/v) Triton X‐100). Complete solubilization of the complex protein samples was achieved through vigorous homogenization using pipetting, followed by desalting with an Amicon Ultra‐0.5 mL 3 K‐NMWL filter (Millipore). A sample of empty PerioPaper (without exudate) was included as a control. The concentration of the complex protein samples was determined using the Bradford method (Bradford 1976). Two micrograms (2 μg) of each complex protein sample, in a final volume of 10 μL, were separately denatured with 2.5 μL of 0.2% RapiGest SF (Waters), reduced with 5 mM DTT (GE Healthcare) at 60°C for 30 min, and alkylated with 15 mM IAA (GE Healthcare). Lastly, the samples were digested with trypsin (Sequencing Grade Modified Trypsin, Promega) at a ratio of 1:100 (w/w) enzyme to protein. After digestion, 1 μL of a 5% (v/v) trifluoroacetic acid aqueous solution was added to the samples. The peptide mixture was centrifuged and dried in a SpeedVac. The resulting peptides were resuspended and desalted using a C18 ZipTip (Millipore, Billerica, MA, USA) according to the manufacturer's instructions and then dried. A final volume of 10 μL was achieved by adding an aqueous solution containing 0.1% formic acid to obtain a final concentration of 200 ng/μL.

### 2.3 Microbiome Analysis

The V3–V4 region of the 16S rRNA gene was amplified using specific primers and PCR conditions (Klindworth et al. 2013). After library preparation, DNA concentrations from biofilm samples were quantified and sequenced, producing paired‐end sequences of 250 bp using the Illumina MiSeq platform. Briefly, the bioinformatics analysis was performed using QIIME2 (Bolyen et al. 2019) software, with data obtained from the demultiplexed FASTQ files from the sequencing resource. After removal of primers (using q2‐cutadapt) (Martin 2011), the amplicon sequence variants (ASVs) were aligned with MAFFT, and taxonomy was assigned to the ASVs using the class sklearn‐naïve Bayes feature classifier trained against the Expanded Human Oral Microbiome (eHOMD, V15.2) (Chen et al. 2010). Alpha and beta‐diversity metrics were estimated. Differences in alpha diversity (within‐sample differences) were assessed using paired differences and linear mixed effects (q2‐longitudinal). For beta diversity (sample differences), group differences were measured with PERMANOVA and PERMDISP tests (q2‐diversity). The impact of time and treatment on the analysis was assessed using the longitudinal distance q2 in pairs. The core microbiome (species present in at least 75% of samples in a group) was calculated using key features from the q2 feature table and visualized in a species‐level table with PhyloToAST (Lin and Peddada 2020). Differential abundance between species was calculated using ANCOM‐BC, considering treatment as an independent variable.

### 2.4 Mass Spectrometry and Metaproteomic Analysis

The mass spectrometry analyses from peri‐implant fluid peptides were conducted using the nanoElute nanoflow chromatographic system (Bruker Daltonics), coupled online to a hybrid trapped ion mobility spectrometry‐quadrupole time‐of‐flight mass spectrometer (timsTOF Pro, Bruker Daltonics). An aliquot (1 μL) of the fluid sample, equivalent to 200 ng of digested peptides, was injected into an Aurora 2 C18 trap column (ionOpticks). The column was online coupled to the timsTOF Pro with a CaptiveSpray ion source (Bruker Daltonics). Liquid chromatography–tandem mass spectrometry (LC–MS) analysis was performed using the PASEF method, which allows for parallel accumulation and fragmentation of ions (Demichev et al. 2022). Data processing, protein identification, and relative quantification analyses were conducted using MaxQuant Software (Version 2.1.3.0). The files generated during processing and searching within UniProt's *Homo sapiens* protein database were used for subsequent analysis with the aid of Perseus Software (Version 2.0.3.0). Only proteins identified in more than 90% of the 12 biological replicates from at least one sample group were considered for statistical analysis, which was performed using MetaboAnalyst Software 5.0 (Xia et al. 2012).

Processing parameters included carbamidomethylation of cysteine as a fixed amino acid modification, while methionine oxidation and N‐terminal acetylation were considered variable modifications. Trypsin was used as the proteolytic enzyme, allowing for a maximum of two cleavage errors. A maximum false discovery rate (FDR) of 1% was employed for peptide and protein identification, with at least one unique peptide required for protein identification. All proteins were identified with a confidence level of ≥ 95% using the MaxQuant algorithm and searches within the UniProt human proteome and Human Oral Microbiome Database (eHOMD) (Chen et al. 2010).

## 3 Results

### 3.1 Clinical and Demographic Data

The mean age of patients included in the study was 51.33 years, with 75% being female. Cancer types affecting the orbital region included melanoma, rhabdomyosarcoma, squamous cell carcinoma (SCC), basal cell carcinoma (BCC), and basaloid squamous cell carcinoma (BSCC), a subtype of SCC. Most patients were treated with a combination of chemotherapy and radiotherapy (Table 1). All titanium implants were from the same brand and model (Prosthesis System Connection, 3.75 × 5 mm Porous). The screwed transcutaneous abutments were also the same model as all implants. The prosthesis retainers were magnetic, either directly attached to the abutment in four patients or mounted on a bar in eight patients. The prosthesis itself was composed of silicone and acrylic resin and was connected to the implant in two different ways: via a metal bar with a magnet or through individual magnets fixed by pillars directly onto the implant.

**TABLE 1:** Clinical and demographic data of the study participants.

Code	Region	Age	Sex	PMH	CT/RT
1	LO	51	F	Melanoma	N
2	RO	60	F	Melanoma	N
3	RO	25	F	Rhabdomyosarcoma	Y
4	RO	36	F	Rhabdomyosarcoma	Y
5	LO	55	M	Basal cell carcinoma (BCC)	Y
6	RO	57	F	Basaloid squamous cell carcinoma (BSCC)	Y
7	RO	57	F	Basal cell carcinoma (BCC)	Y
8	LO	56	F	Squamous cell carcinoma (SCC)	Y
9	RO	73	M	Squamous cell carcinoma (SCC)	Y
10	RO	40	M	Melanoma	N
11	LO	42	F	Squamous cell carcinoma (SCC)	Y
12	RO	63	M	Squamous cell carcinoma (SCC)	Y

Abbreviations: BCC, basal cell carcinoma; BSCC, basaloid squamous cell carcinoma; CT/RT, Combined chemotherapy and radiotherapy; D, disease; H, health; LO, left orbit; N, No; PMD, past medical history; RO, right orbit; SCC, squamous cell carcinoma; Y, Yes.

### 3.2 Characterization of the Dysbiotic Bacterial Community in Diseased Craniofacial Implants

**FIGURE 2:**
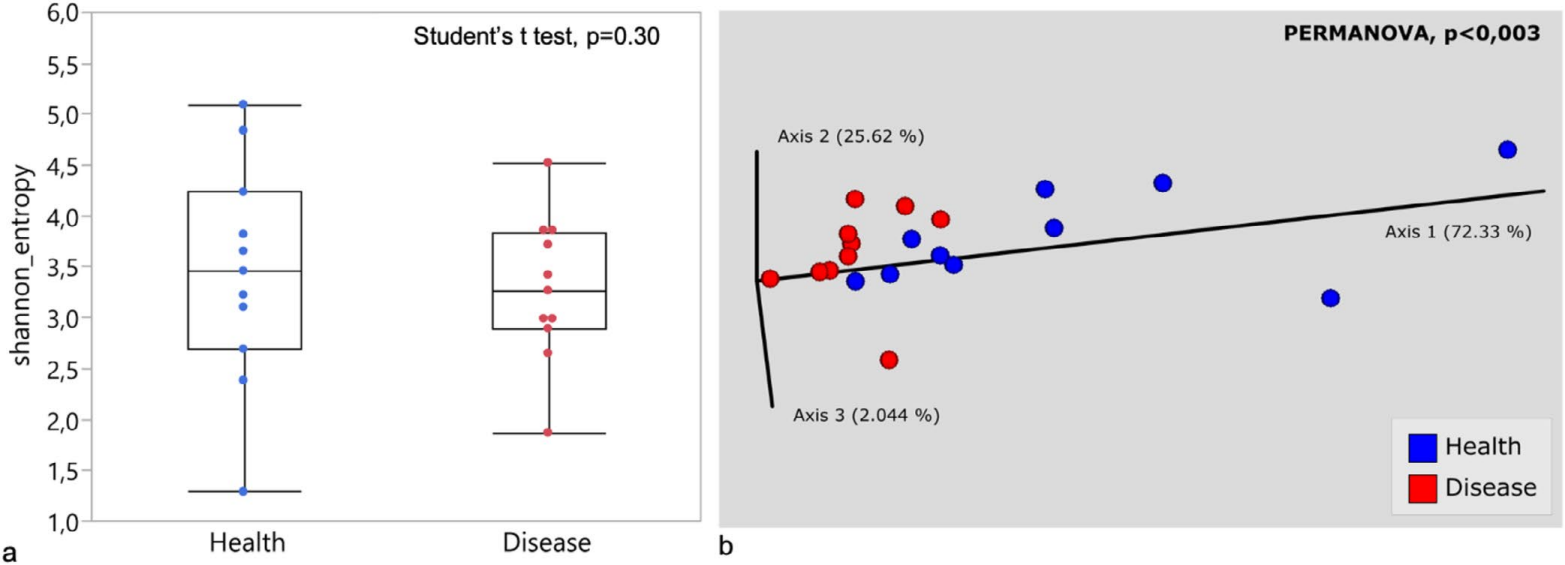
(a) Alpha diversity measured by the Shannon index for Health and Disease groups (Student's *t*‐test). (b) Beta diversity calculated using compositional tensor factoring (CTF) and visualized through principal coordinate analysis (PCoA) for the healthy and disease groups.

Although no differences were observed in alpha diversity analysis using the Shannon metric (Figure 2A), healthy and diseased CI displayed distinct microbial communities in the beta‐diversity analysis (*p* = 0.003). Principal coordinate analysis revealed that the microbial communities in the diseased group were more tightly clustered (Figure 2B).

**FIGURE 3:**
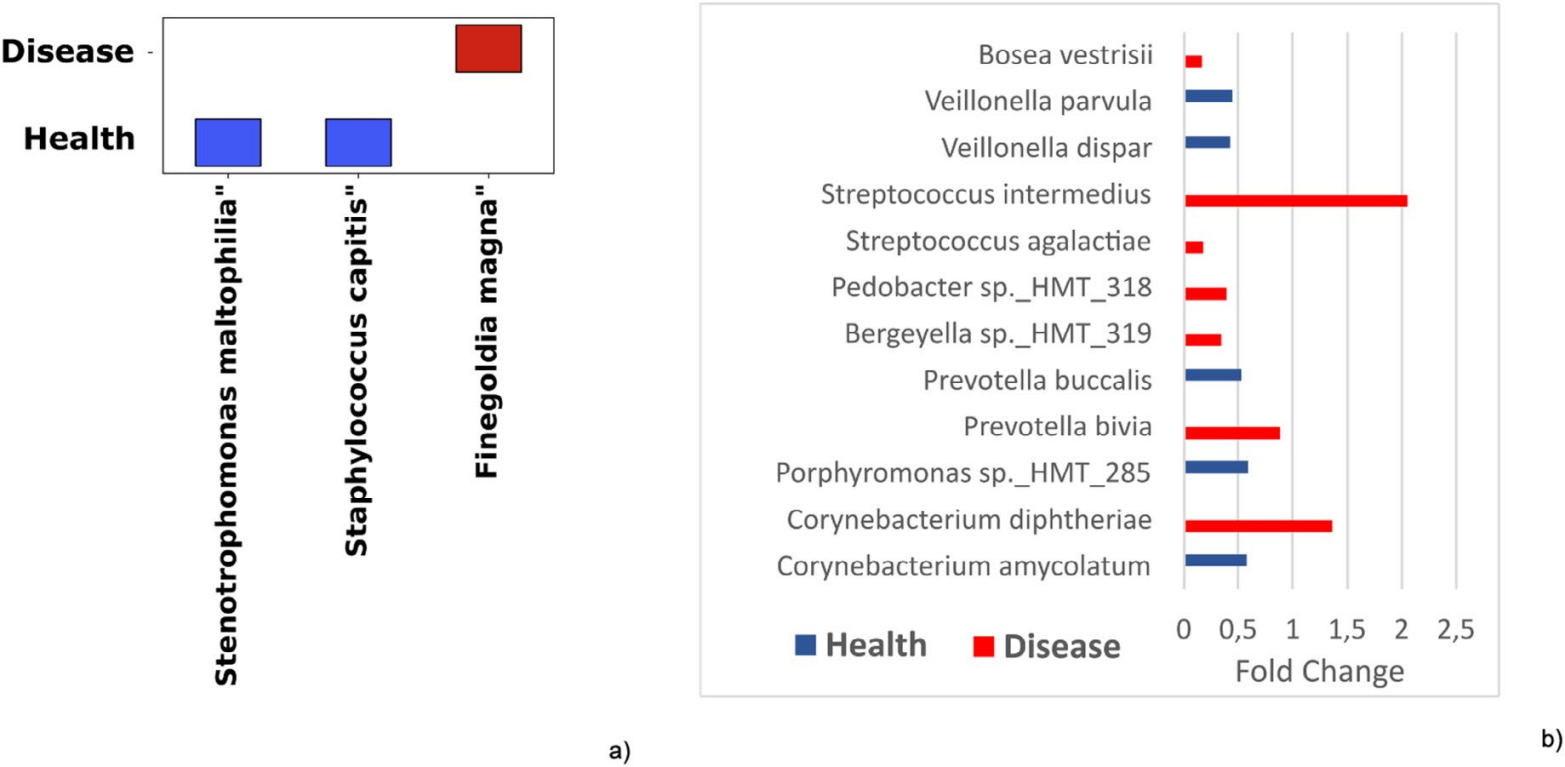
(a) Core microbiome in the test and control groups, with species present in at least 75% of samples from each group considered part of the core microbiome. (b) Species differentially abundant between diseased and healthy groups, as identified by ANCOMBC software, with differences shown as log‐fold changes. Red bars represent the most abundant species in the diseased group, while blue bars indicate the most abundant species in the healthy group.

Core microbiome analysis revealed that *Stenotrophomonas maltophilia* and *Streptococcus capitis* were more prevalent in the healthy group, while *Finegoldia magna* was more prevalent in the diseased group (Figure 3A). In terms of differential abundance, *Corynebacterium diphtheriae* and *Prevotella bivia* were significantly more abundant in the diseased implants, with *Streptococcus intermedius* showing a twofold increase, along with *Pedobacter* sp., *Bergeyella* sp., *Streptococcus agalactiae* , and *Bosia vestrisii*. Conversely, certain species were more abundant in the healthy group, including *Corynebacterium amycolatum* , *Porphyromonas* sp., *Prevotella buccalis* , *Veillonella parvula* , and *Veillonella dispar* (Figure 3B).

### 3.3 The Disease Group is Also Associated With a Distinct Local Inflammatory Signature

**FIGURE 4:**
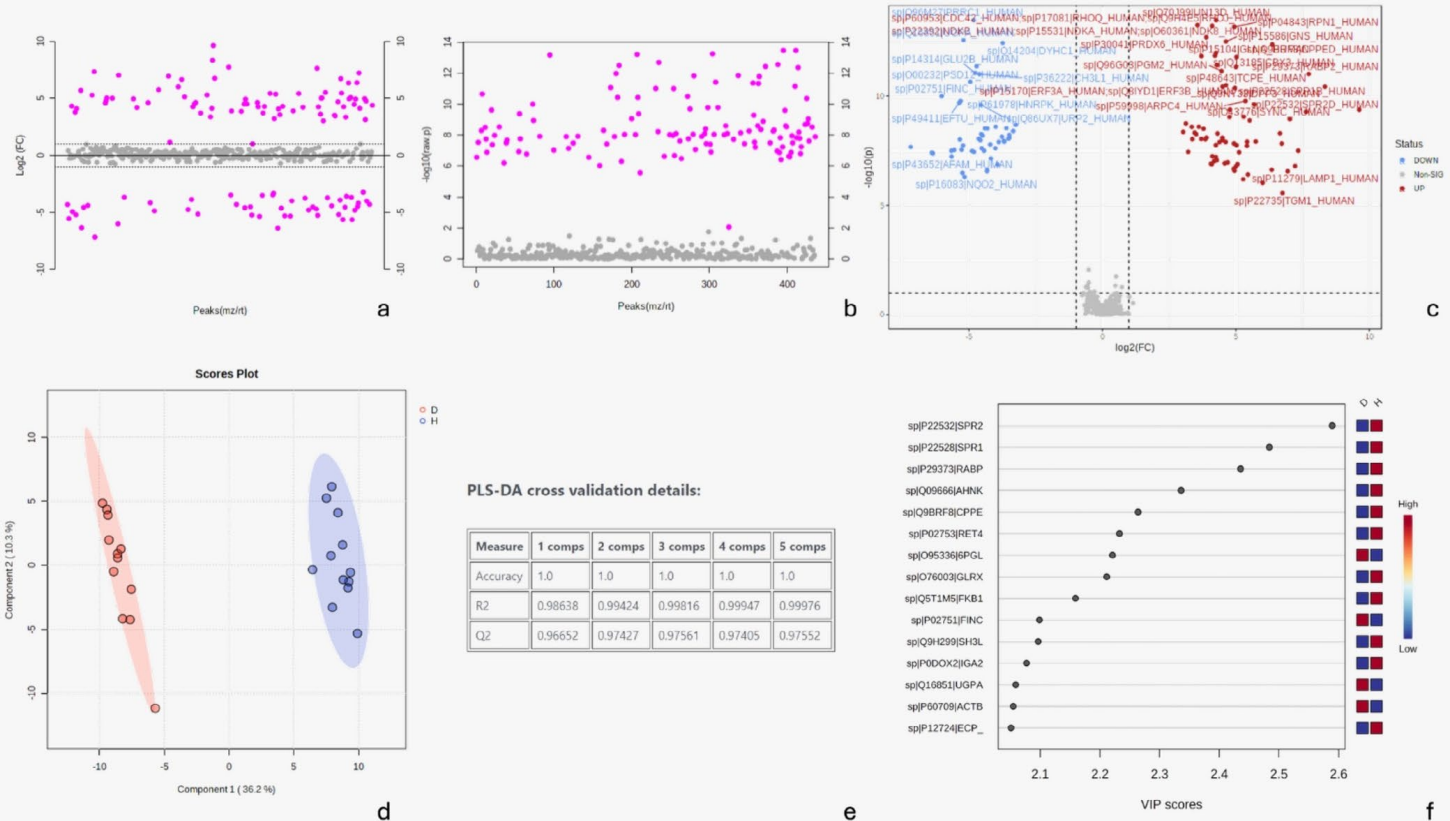
(a) *T*‐test (*p* ≤ 0.05) on a logarithmic scale. (b) Fold‐change analysis on a logarithmic scale with *T*‐test (*p* ≤ 0.05). Nonsignificant abundance is shown in gray, while significant ones are highlighted in pink, comparing Groups D to S. (c) Volcano plot (*p* ≤ 0.05, adjusted) and fold‐change (> 2). (d) Variance analysis—VIP score graph. (e) Multivariate analysis (PLS‐DA). (f) Accuracy—theoretical versus observed, considering outliers. (g) Importance of the projected variable (VIP score). Values obtained from PLS‐DA analysis. The colored boxes on the right represent the relative abundance of corresponding metabolites in each group (D and H).

Metaproteomic analysis identified 147 proteins that were differentially expressed between healthy and diseased CI (*p* ≤ 0.05) (Figure 4A–C, Tables S1–S4). Partial Least Squares Discriminant Analysis (PLS‐DA) revealed clear group separation, with *R* ^*2*^ and *Q* ^*2*^ values ≥ 0.9, indicating an optimal model fit (Figure 4D,E). The host proteomic analysis showed distinct protein abundance patterns, with Components 1 and 2 explaining 46.5% of the variance in the model.

**FIGURE 5:**
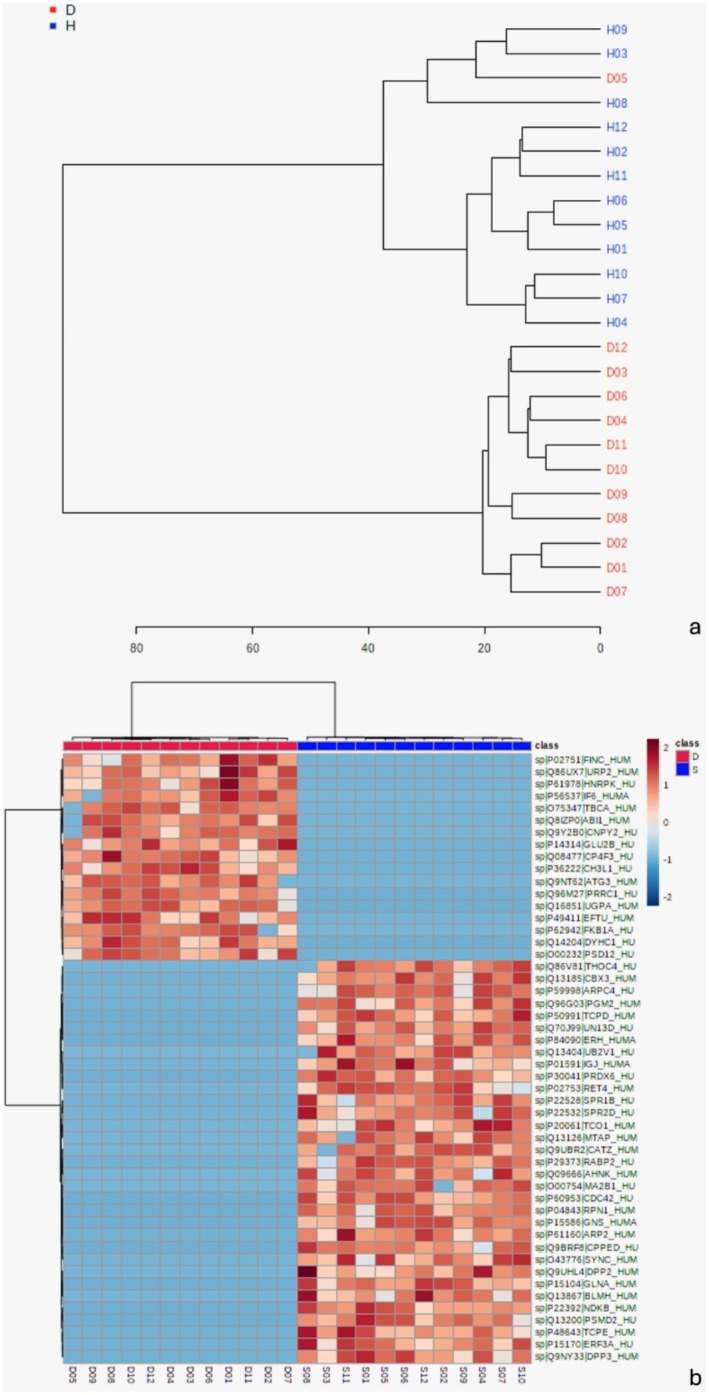
(a) Clustering results shown as a dendrogram (distance measured using Euclidean method and clustering algorithm using Ward. D). (b) Heatmap based on differentially abundant proteins (*p* ≤ 0.05, FDR adjusted). Clustering results are shown as a heatmap of the 50 most abundant proteins.

The 15 proteins with the greatest differences in abundance between the groups are highlighted in the VIP scores chart (variable importance in projection), showing both shared and unique proteins for each group under this experimental condition (Figure 4F). When clustering the 50 proteins with the largest fold changes, adjusted for FDR (*p* ≤ 0.05), a dendrogram reveals decreased protein abundance in the disease group and increased abundance in the health group, impacting fold change values directly (Figure 5, Tables S2 and S3).

In terms of biological processes, clustering analysis revealed proteins involved in homeostatic maintenance within the health group, including roles in tissue, cellular, and biological reactions. Key proteins and functions identified were CDC24 (ATP binding, cytoplasm mediator), ARPC4 (DNA repair), THOC4 (mRNA transport), CBX‐3 (protein transport), AKNK (neuronal cell differentiation), MA2B1 (carbohydrate catabolism), and ARP2 (ATP binding with nucleation‐promoting factor, NPF), along with other regulatory proteins aligned with tissue response pathways.

In contrast, the diseased group showed higher abundance of proteins linked to wound healing and coagulation, such as FINC, INF6, and URP2 (homeostasis regulation). Additional proteins included PSD‐12 (protein repair), HNRPK (mRNA transport, acid‐binding, and immune influence), and EFTU (innate immunity regulation). Inflammatory proteins, such as CNPY2 (neuritis marker) and IL3 (CH3L1), a preinflammatory factor and tumor marker, were also more prevalent, along with immune and wound healing modulators CP4F3 (cytochrome P450) and FKB1A (TGF‐B receptor blocker), which regulate inflammation and immune responses.

**FIGURE 6:**
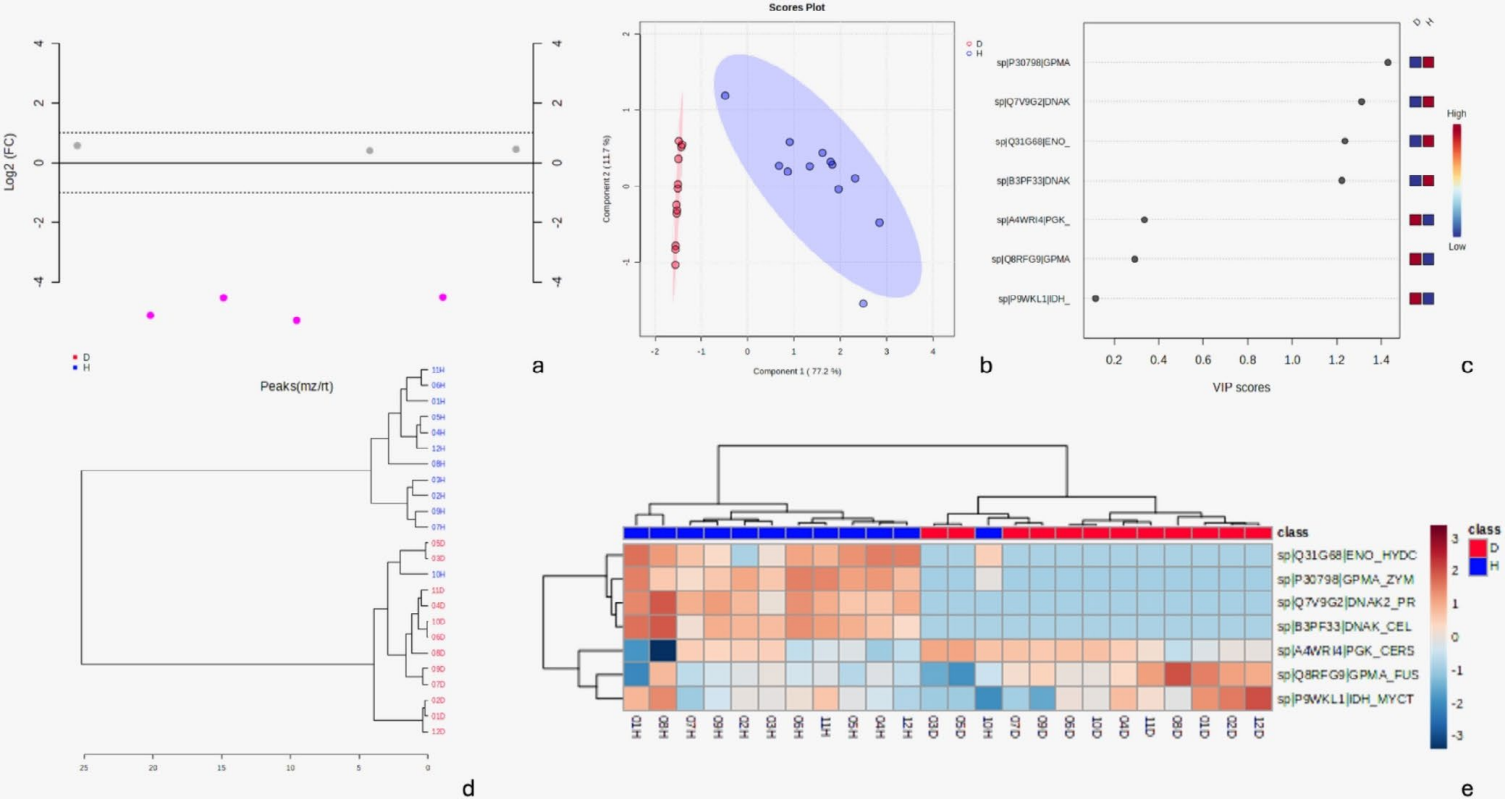
(a) The fold‐change graph shows the level of significance, with significant proteins in pink and nonsignificant proteins in gray, near zero. (b) PLS‐DA demonstrates the separation of groups in 90% of the analyzed proteins, with the disease group showing lower diversity and the health group exhibiting greater diversity. (c) The VIP score reveals that the first four proteins were highly abundant in the health group, while the last three were more abundant in the disease group. (d) Clustering results are shown as a dendrogram (distance measured using the Euclidean method and clustering algorithm using Ward. (e) Heatmap based on differentially abundant proteins (*p* ≤ 0.05, FDR adjusted).

Regarding bacterial proteins, analysis of human‐to‐bacterial peptide ratios indicated a lower proportion of bacterial peptides in the disease group compared to the health group. Although only a few proteins were significantly more abundant (Figure 6A, Table S4), PLS‐DA analysis revealed a clear separation between groups for 90% of the proteins analyzed, with lower diversity in the disease group and higher diversity in the health group (Figure 6B). In the disease group, three bacterial proteins were most abundant in disease samples, including phosphoglycerate kinase, 2,3‐bisphosphoglycerate‐dependent phosphoglycerate mutase (glycolysis and carbohydrate degradation protein from *Fusobacterium nucleatum* ), and *Mycobacterium tuberculosis* oxidoreductase isocitrate dehydrogenase‐NADP, which also appeared at lower abundance in the health group, suggesting their potential role in dysbiosis. A heatmap of bacterial proteins further illustrated distinct clustering between health and disease (Figure 6E).

The VIP score graph (Figure 6C) highlights the functional roles of key proteins and their associated microorganisms. Notably, *Zymomonas mobilis* 's P30798/GPMA is involved in carbohydrate catalysis and glycolysis, *Prochlorococcus marinus* 's Q7V9G2/DNAK has ATP and nucleotide shielding functions for stress response (also observed in *Cellvibrio japonicus* ), and *Caetibacter sphaeroides'*s B3PF33/DNAK shows catalytic activity—all more abundant in the health group.

## 4 Discussion

Extensive research has been conducted on the microbiome in oral implantology for many years (Corrêa et al. 2019; Pimentel et al. 2018). However, in the field of CI‐retained prostheses, no studies have yet focused on identifying the microbiome/proteome around implants and comparing healthy versus diseased areas, making this study pioneering in this regard. By identifying the microbial composition and inflammatory pathways, it may be possible to find ways to prevent complications and implant failures and promote peri‐implant health, ultimately enhancing patients' quality of life. Our findings revealed a dominance of certain species in diseased areas, potentially linked to the etiopathogenesis of facial skin inflammation, such as *Streptococcus intermedius* , *Corynebacterium diphtheriae* , and *Prevotella bivia* , accompanied by a distinct host and bacterial proteome profile with increased abundance of inflammation‐related proteins.

Among the microbial species found in diseased implants, such as *Finegoldia magna* , *Streptococcus intermedius* , and *Prevotella bivia* , there are established associations with inflammation and bone infections related to metallic prostheses (Johansson et al. 2021; Samantaray et al. 2020; Söderquist et al. 2017), which may threaten the long‐term maintenance of the implant due to their pathogenicity and virulence. Additionally, previous studies have shown that *Corynebacterium diphtheriae* can invade tissues and the bloodstream and cause infections in other areas of the body (Ott et al. 2010; Ott et al. 2022; Peixoto et al. 2016). Therefore, the microbial community in diseased implants appears to threaten not only the local transmucosal interface but also has the potential to spread throughout the body and exert systemic effects. In this context, the presence of a diseased TRCI in a susceptible individual (such as those presenting a history of head–neck cancer) could increase the risk for systemic impact and decrease the quality of life.

Additionally, the microbial profile of TRCI implants differs significantly from intraoral implants, despite similar soft/hard tissue histology and implant materials. Previous studies have shown that healthy intraoral implants harbor an enriched community of *Streptococcus* sp. and *Corynebacterium* sp., with a core microbiome comprising *Actinomyce*s, *Capnocytophaga*, *Rothia, Neisseria*, *Corynebacterium* sp., and *Streptococcus* (Dabdoub et al. 2013; Di Spirito et al. 2024; Pimentel et al. 2018). In our study, even healthy TRCI implants exhibited higher abundances of *Veillonella*, *Porphyromonas*, and *Prevotella*, genera typically associated with periodontal and peri‐implant diseases. This finding suggests that the environment is the primary determinant of the microbiome composition, rendering intra‐ and extraoral peri‐implant niches incomparable. Supporting this, a recent study (Dutra et al. 2024) identified *Corynebacterium durum* and *Delftia acidovorans* as discriminative features of healthy intraoral implants. Interestingly, this study also highlighted that health is associated not only with specific microbial species but also with a reduced inflammatory cytokine profile, particularly elevated IL‐4 levels, whereas inflamed implants show increased proinflammatory mediators. In conclusion, the environment's role in shaping the microbial community is inseparable from the local host response, as further evidenced by our proteomic analysis.

Moreover, the metaproteome reveals that the diseased transmucosal region exhibits a distinct functional bacterial profile, with proteins that are not necessarily enriched by the most abundant community members but still capable of influencing pathogenicity. Among the three bacterial genes found to be more abundant in disease, the glycolytic enzyme phosphoglycerate was reported in *Streptococcus pneumoniae* to play a role in evading the host immune response via complement system attack, thereby causing local infections (Blom et al. 2014). This finding is particularly relevant since another *Streptococcus* species was also found to be more abundant in the disease group. Similarly, the 2,3‐bisphosphoglycerate‐dependent phosphoglycerate mutase protein from *F. nucleatum* , a well‐known pathogen associated with oral, head and neck cancers (Al‐Hebshi et al. 2017; Audirac‐Chalifour et al. 2016), showed increased abundance at most diseased sites. While the role of *F. nucleatum* in extraoral diseases is not fully defined, and despite encoding canonical virulence factors (Zanzoni et al. 2017), literature suggests its mechanistic role as a “passenger” in tumorigenesis (Tjalsma et al. 2012). As we observed no significant differences in *F. nucleatum* abundance between groups but noted increased functional activity, we can suggest that *F. nucleatum* in disease may exhibit a behavioral influence rather than abundance‐driven effects, and other microorganisms, such as fungi, can potentially contribute to site virulence and inflammation, which were seen in previous reports (Li et al. 2023; Wu et al. 2015).

In health, the proteome was more closely associated with metabolic signatures of keratinocytes and skin‐related proteins. However, the set of identified proteins in disease indicated that this altered microbial community is not only associated with TRCI but also reflects an imbalance in the host response and a suppression of immune activity. Consistent with previous literature, the disease group showed increased levels of the TGF‐B receptor (FKB1A), which has been linked to tumor immune cell infiltration in carcinoma (Li et al. 2022), and CH3L1, associated with myocardial fibrosis (Sun et al. 2023). These findings indicate patterns of an unbalanced immune response and impaired wound healing.

Furthermore, several proteins identified in our study have been previously reported in the literature to possess diagnostic value for tumors. The increased abundance of the CD5 antigen‐like apoptosis inhibitor in the disease group may suggest an aggravation of local inflammation, as this protein is associated with diagnostic and prognostic markers in various cancer‐related clinical scenarios (Choi et al. 2021; Okanoue et al. 2022; Yamazaki et al. 2014). Additionally, Immunoglobulin lambda (Igλ), including IGLC7, was enriched in our disease analysis and has been reported in the literature as correlated with cervical cancer tissues (Wang et al. 2023). The increased abundance of MUC16 in the disease group is also noteworthy, as this aberrant glycoprotein is highly expressed in several solid tumors and different types of cancers (Lakshmanan et al. 2017; Lee et al. 2024; Lin et al. 2024; Rao et al. 2015). Normally, MUC16 protects the apical surface of epithelia, but its overexpression on solid tumors promotes tumor progression, metastasis, and immune escape by directly suppressing host defense cells (Lee et al. 2024; Rao et al. 2015). In epithelial ovarian cancer cells, MUC16 plays a key role in tumor growth and metastasis (Lee et al. 2024). Given the role of epithelial cells in driving malignancy in the cancer types observed in this study, further investigation is warranted into the relationship between MUC16, skin reactions, and head and neck cancer.

This study presents certain limitations. First, the limited number of patients and the relative heterogeneity in age, sex, and cancer history among participants must be acknowledged. Given the highly specific condition under investigation and the strict inclusion criteria for allocation into health or disease groups, it was not feasible to include a larger patient cohort. Additionally, it is important to note that factors potentially influencing the biofilm–host relationships—such as smoking, cancer treatment, psychological stress, comorbidities, and possible contamination by other infectious agents (e.g., fungi)—were not assessed. However, the intraindividual comparison of sites with varying peri‐implant conditions helped reduce bias, as differing microbiological and inflammatory profiles within the same individual may better control for inter‐individual variability. As this is the first study to evaluate both the microbiome and proteomic profile in oncology patients with extraoral implants, future research should aim to incorporate these additional variables for a more comprehensive analysis and to validate our findings. Anyway, taken together, the results provide novel insights into host–microbial interactions surrounding CI and highlight potential avenues for improving clinical outcomes and ensuring successful rehabilitation.

## Author Contributions

**Daniela Lattuf Cortizo:** investigation, writing – original draft, writing – review and editing, visualization, formal analysis, data curation, methodology. **Renato C. V. Casarin:** writing – original draft, writing – review and editing, investigation, methodology, formal analysis. **Hélvis E. S. Paz:** writing – original draft, writing – review and editing, methodology, formal analysis, data curation. **Camila S. Stolf:** writing – review and editing, methodology, formal analysis, data curation. **Mabelle F. Monteiro:** methodology, visualization, formal analysis, writing – review and editing. **Mônica T. V. Labate:** writing – review and editing, formal analysis, methodology, data curation. **Márcio Z. Casati:** investigation, writing – review and editing, supervision. **Luciano Lauria Dib:** supervision, project administration, resources, investigation, writing – review and editing.

## Conflicts of Interest

The authors declare no conflicts of interest.

## Supporting information


Tables S1–S4.

## Data Availability

The data that support the findings of this study are openly available in Sequence Read Archives (SRA) of the Center for Biotechnology at https://www.ncbi.nlm.nih.gov/sra, reference number PRJNA1073959.
